# Effects of Ubiquinol-10 on MicroRNA-146a Expression In Vitro and In Vivo

**DOI:** 10.1155/2009/415437

**Published:** 2009-04-16

**Authors:** Constance Schmelzer, Mitsuaki Kitano, Gerald Rimbach, Petra Niklowitz, Thomas Menke, Kazunori Hosoe, Frank Döring

**Affiliations:** ^1^Institute of Human Nutrition and Food Science, Molecular Nutrition, Christian-Albrechts-University of Kiel, Heinrich-Hecht-Platz 10, 24118 Kiel, Germany; ^2^Frontier Biochemical and Medical Research Laboratories, Kaneka Corporation, Takasago, Hyogo, Japan; ^3^Institute of Human Nutrition and Food Science, Food Science, Christian-Albrechts-University of Kiel, Hermann-Rodewald-Street 6, 24098 Kiel, Germany; ^4^Vestische Kinder-und Jugendklinik Datteln, Universität Written/Herdecke, Dr.-Friedrich-Steiner Street 5, 45711 Datteln, Germany; ^5^Functional Food Ingredients Division, Kaneka Corporation, Osaka, Japan

## Abstract

MicroRNAs (miRs) are involved in key biological processes via suppression of gene expression at posttranscriptional levels. According to their superior functions, subtle modulation of miR expression by certain compounds or nutrients is desirable under particular conditions. Bacterial lipopolysaccharide (LPS) induces a reactive oxygen species-/NF-κB-dependent pathway which increases the expression of the anti-inflammatory miR-146a. We hypothesized that this induction could be modulated by the antioxidant ubiquinol-10. Preincubation of human monocytic THP-1 cells with ubiquinol-10 reduced the LPS-induced expression level of miR-146a to 78.9 ± 13.22%. In liver samples of mice injected with LPS, supplementation with ubiquinol-10 leads to a reduction of LPS-induced miR-146a expression to 78.12 ± 21.25%. From these consistent in vitro and in vivo data, we conclude that ubiquinol-10 may fine-tune the inflammatory response via moderate reduction of miR-146a expression.

## 1. Introduction

MicroRNAs (miRs) are endogenous ∼22 nucleotide noncoding RNAs which suppress gene expression at posttranscriptional levels by binding to the 3′-untranslated region of specific mRNA targets through base pairing [1]. A number of recent studies reveal that miRs have critical functions in key biological processes such as cell proliferation and cell death during development [2], fat metabolism [3, 4], or insulin secretion [5]. Expression profiling approaches implicated a differential expression of several miRs in cancer cells [1]. Regulated expression of miRs is scarcely understood but is of special interest in the context of innate immunity and inflammation [6]. Expression profiling of more than 200 miRs in human monocytic THP-1 cells revealed that miR-146a is substantially inducible by LPS or other Toll-like receptor ligands [7]. Moreover, promoter analysis of the miR-146a gene suggests a role of NF-*κ*B in LPS-dependent upregulation of miR-146a [7].

Modulation of the LPS → reactive oxygen species → NF-*κ*B signalling pathway and dependent miR-146a expression could be an approach in order to fine-tune the inflammatory response. The importance of such a modulation is of special interest because the balance between pro- and anti-inflammatory signals is critical in certain inflammatory diseases such as atherosclerosis. Here we hypothesized that the reduced form of Coenzyme Q_10_ (QH_2_, ubiquinol-10) affects LPS-inducible expression of the anti-inflammatory miR-146a. The hypothesis was based on functions of CoQ_10_ as a potent antioxidant and putative modulator of inflammation via gene expression [8, 9]. In the present work, we tested our hypothesis using an in vitro and an in vivo model. Initially, we utilized the human monocytic cell line THP-1 as a well-established model of LPS-induced miR-146a expression and applied ubiquinol-10. Additionally, liver samples of LPS-injected mice supplemented with ubiquinol-10 were used to strengthen the hypothesis that ubiquinol-10 has a relevance in fine-tuning LPS-induced miR-146a expression levels.

## 2. Materials and Methods

### 2.1. Reagents

Ubiquinol-10 aqueous solution (PEG-60 hydrogenated castor oil, ubiquinol-10, glycerol, water) and corresponding vehicle (no ubiquinol-10 supplement) were purchased from KANEKA Corporation, Japan. Lipopolysaccharide (LPS, *E. coli* O55:B5) was obtained from Sigma-Aldrich (Germany).

### 2.2. Cell Culture

 THP-1 cells were routinely cultivated in RPMI medium 1640 supplemented with 10% FCS and 1% antibiotics (penicillin/streptomycin) in a humidified incubator containing 5% CO_2_ at 37°C. Twenty four hours before preincubation, cells were plated at a density of 1 × 10^6^ cells per well in a 6-well plate. Subsequently, cells were preincubated with 10 *μ*M ubiquinol-10 or the respective vehicle control. After 24 hours, cell culture medium was removed and fresh LPS-containing medium (1 *μ*g/mL) was added for 4 hours. Finally, cells were collected in Qiazol-lysis buffer (Qiagen, Germany) for isolation of total RNA or alternatively scraped in Phosphate Buffered Saline (PBS) for HPLC-analysis.

### 2.3. Animals

Male C57/BL6J mice (10–12 weeks old, 25 g weight) were purchased from Charles River Lab., Inc., Japan. Animals were separated into two groups: (1) intervention group (*n* = 6), which was given a diet enriched with ubiquinol-10 (QH_2_, 250 mg/kg/d) for one week and (2) the control group (*n* = 6), which received a diet prepared by using corn oil in equal proportions to 1% (v/w) of the diet as a vehicle. In other respects all animals were maintained on a standard laboratory diet (powdered CE-2, CREA Japan) and housed under conditions at 22 ± 2°C with a 12-hour light/dark cycle. Food intake and body weight were monitored daily but indicated no relevant differences between animals. After the 7-day supplementation or control diet period, an intraperitoneal injection (1 mg/kg BW) of lipopolysaccharide (LPS, *E. coli*, O55:B5, Sigma-Aldrich, Japan) was administered for further 4 hours. However, for HPLC-experiments, only saline-injected mice were used. In all other aspects, animal treatment was identical. Subsequently, all mice were sacrificed, and liver samples were collected and stored at −80°C in RNAlater Storage Solution (Qiagen, Japan) until use.

### 2.4. Cytotoxicity of THP-1 Cells

**Figure 1:**
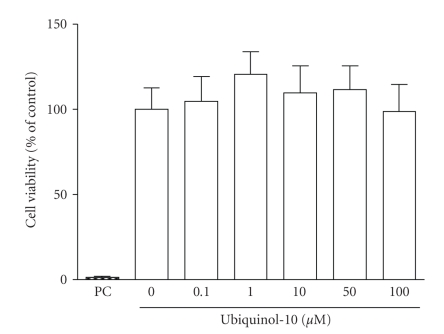
Effect of ubiquinol-10 on viability of THP-1 monocytes. THP-1 cells were treated with medium, 0.1–100 *μ*M ubiquinol-10, or 10% DMSO (PC, positive control) for 24 hours. Afterwards, cell viability was determined based on ATP measurements. The viability of medium control was set to 100%, and the other values were referenced to it. Data are means ± SD of three independent experiments performed in triplicate.

 Determination of cell viability was performed using the Cell-Titer Glo Luminescent Assay. Thus, the total ATP levels were measured as an index of the viable cell number. The luminescence was detected on a GloMax (Promega). Data (Figure 1) are means ± SD of three biological experiments performed in triplicate.

### 2.5. Determination of CoQ_10_ In Vitro and In Vivo

The different treated cells of each well were washed with Phosphate Buffered Saline (PBS) and counted with Trypan blue at −80°C before measurement of cellular CoQ_10_. Liver homogenates of 10 QH_2_-supplemented or nonsupplemented mice (5 from each group, resp.) were stored at −80°C (in 0.9% sodium chloride, 10 mg/mL) until further analysis. The method is based on high-pressure liquid chromatography (HPLC) with electrochemical detection and internal standardisation using ubihydroquinone-9 and ubiquinone-9 as standards and is described elsewhere [10]. In brief, as internal standard 15 pmol of ubihydroquinone-9 in 50 *μ*L ethanol were added to a 100 *μ*L monocyte or liver homogenate suspension. The cells and homogenates were disintegrated by adding of 300 *μ*L of cold methanol. Subsequently, the sample was mixed for 1 minute, and the suspension was immediately extracted with 500 *μ*L hexane after mixing for further 2 minutes. After centrifugation (1000 xg, 5 minutes, 4°C), 300 *μ*L of the supernatant were transferred to a separate tube and dried under a stream of argon. Finally, the dried residue was redissolved in 40 *μ*L ethanol and injected into the HPLC system. For each liver homogenate sample, the analyzed CoQ_10_ concentration was related to its respective protein level.

### 2.6. Protein Quantification of Liver Homogenate Solutions

For calculation of differences in sample preparation, protein concentration was determined in liver homogenate samples (in *μ*g/ml). Thus, homogenate samples were collected into NET-buffer (50 mM TRIS [pH 7.5], 150 mM NaCl, 1 mM EDTA [pH 8.0], 0.5% NP-40). In each case, homogenate solutions were treated with ultrasonics (“*n*” vs. “*m*”) and then centrifuged by 14.000 rpm at 4°C for 20 minutes. The protein concentration was determined by the Bradford method according to the manufacturer's instructions.

### 2.7. RNA Isolation and Quantitative RT-PCR of THP-1 Cells

Total RNA was isolated by using the miRNeasy Isolation Kit (Qiagen, Germany), and cDNA was converted by the TaqManMicroRNA Reverse Transcription Kit (Applied Biosystems). miRNA-146a expression was measured and quantified by using the TaqManMicroRNA Assays (Applied Biosystems) according to the manufacturer's protocol and normalised by snoRNA202 (Applied Biosystems). Quantitative RT-PCR reaction was performed on an Applied Biosystems 7300 Real-Time PCR System.

### 2.8. RNA Isolation of Liver Samples and Quantitative RT-PCR

Total RNA was isolated with Qiazol lysis reagent obtained with the miRNeasy Isolation Kit (Qiagen, Germany). cDNA was converted by the TaqMan MicroRNA Reverse Transcription (RT) Kit (Applied Biosystems). The RT reaction product was diluted 10 times in water and subsequently used for RT-PCR amplification of miRNA-146a by using the TaqMan MicroRNA Assays (Applied Biosystems) according to the manufacturer's protocol and normalized by snoRNA202 (Applied Biosystems). A 6-fold total RNA-dilution series from a control-treated (+LPS) mouse liver served as standard to ensure a linear range of the amplification. Quantitative RT-PCR reaction was performed on an Applied Biosystems 7300 Real-Time PCR System.

### 2.9. Statistics

 Results were analyzed by an unpaired, two- or one-sided Student's *t*-test using SPSS 11.5 for Windows and GraphPad Prism 4.0 software. *P*-values less than or equal to .05 were considered statistically significant.

## 3. Results and Discussion

### 3.1. No Cytotoxic Effects but Cellular Accumulation of Ubiquinol-10 in the Human Monocytic Cell Line THP-1

To exclude cytotoxic side effects in our experimental set-up, vitality of THP-1 cells was determined after incubation with ubiquinol-10. As shown in Figure 1, incubation of THP-1 cells with increasing ubiquinol-10 concentrations (0.1–100 *μ*M) for 24 hours led to no significant effects on cell vitality. Thus, in THP-1 cells no cytotoxicity was found for ubiquinol-10 at physiological (1.0 *μ*M), supraphysiological (10 *μ*M), and pharmacological (>10 *μ*M) concentrations. The putative effects of extracellular ubiquinol-10 on miR expression depend on its capability to reach cellular concentrations above background level. Therefore, we determined the cellular concentration of CoQ_10_ as a function of medium ubiquinol-10. As shown in Table 1, cellular CoQ_10_ levels arose with increasing extracellular ubiquinol-10 concentrations (0.1–100 *μ*M). As mainly the reduced form of CoQ_10_ can function as an antioxidant [11], we determined the proportion between the oxidized and reduced form of CoQ_10_. Depending on the extracellular ubiquinol-10 concentration, about 75–90% of cellular CoQ_10_ was present in its reduced form (Table 1). Moreover, our previous results have shown that monocytic cells are able to convert oxidized CoQ_10_ effectively into its reduced form [12]. Thus, at physiological and supraphysiological CoQ_10_ levels in the medium (0.1–10 *μ*M), the intracellular CoQ_10_ distribution is clearly in favor of the reduced form (75–90%). Other in vitro and in vivo studies also revealed an intracellular incorporation of CoQ_10_ after supplementation in blood cells, thereby leading, for example, to a reduction of DNA strand breakdowns [11, 13]. Taken together, we were able to increase the cellular ubiquinol-10 concentration without any cytotoxic side-effects in human THP-1 cells.

### 3.2. Ubiquinol-10 Attenuates the LPS-Induced Expression of miR-146a in the Human Monocytic Cell Line THP-1

**Figure 2:**
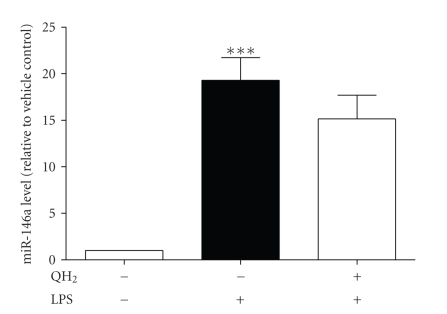
Effect of ubiquinol-10 on LPS-induced miR-146a expression in THP-1 cells. THP-1-cells were preincubated without (−) or with (+) 10 *μ*M ubiquinol-10 for 24 hours. Afterwards, cells were treated without (−) or with (+) 1 *μ*g LPS/mL for 4 hours. After this treatment, total RNA was extracted, converted to cDNA and miRs were assayed by TaqMan-based qRT-PCR. Observed expression levels of miRs in the respective treated cells were normalized to the corresponding levels of the endogenous control (snoRNA202). Data (two-sided *t*-test) are means ± SEM of three biological experiments performed in quadruplicate. *P < .05 versus unstimulated cells. ***P < .001 versus unstimulated cells.

In order to study the effect of ubiquinol-10 on LPS-induced miR-146a expression, appropriate conditions were established. For this purpose, the LPS-induced response of THP-1 cells was examined. Unstimulated THP-1 cells did not secrete relevant amounts of TNF-*α* into the medium (4.22 pg/mg protein ± SD). However, stimulation with 1 *μ*g/mL LPS for 4 hours resulted in an increase of medium TNF-*α* levels (351.94 pg/mg protein ± SD). Thus, we obtained a substantial and anticipated LPS-response of THP-1 cells [14]. As already described in the literature [7], the expression level of miR-146a is upregulated by LPS. As shown in Figure 2, in comparison to unstimulated THP-1 cells, LPS challenge induced a 19-fold induction of miR-146a expression (*P* = .0007). With respect to our results regarding cytotoxicity and cellular accumulation of ubiquinol-10, THP-1 cells were preincubated with 10 *μ*M ubiquinol-10 for 24 hours as an effective dose. This is nearly in accordance to other in vitro studies performed with CoQ_10_ [15, 16] and is only 2-fold higher than CoQ_10_ serum levels after supplementation in humans [17]. Thereafter, cells were stimulated with 1 *μ*g/mL LPS for 4 hours and the resulting steady-state expression level of miR-146a was determined. As shown in Figure 2, preincubation of THP-1 cells with ubiquinol-10 reduced the LPS-induced expression level of miR-146a to 78.9 ± 13.22%. Although these effects were not statistically significant (*P* ≤ .05), the reduced expression levels of miR-146a in the ubiquinol-10-pretreated cells (QH_2_ + LPS) might be a first hint for a fine-tuning mechanism of QH_2_ on miR-146a expression when compared to the high significant induction levels in control cells after LPS-stimulation (Veco LPS).

### 3.3. Accumulation of CoQ_10_ Levels in the Liver of Ubiquinol-10 Supplemented Mice

**Figure 3:**
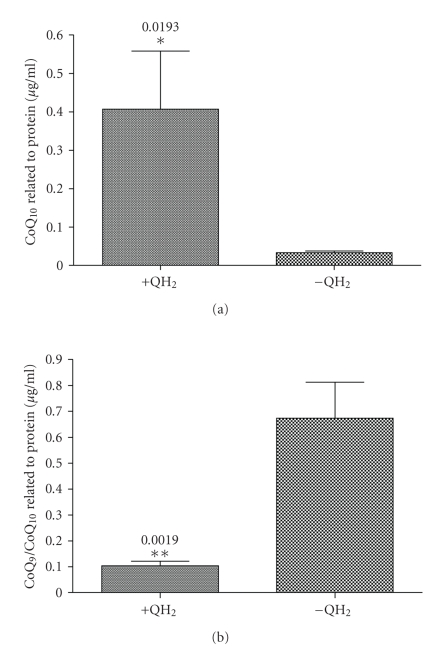
Effect of ubiquinol-10 concentration (a) on CoQ_10_ levels and (b) the CoQ_9_/CoQ_10_ ratio in liver tissues of mice. Mice were fed with ubiquinol-10 (250 mg/kg/d) or a respective control diet (no ubiquinol-10 supplement) for 7 days followed by a sublethal intraperitoneal injection with saline (0.9% NaCl, LPS negative control) for further 4 hours. Thereafter, liver samples were collected, homogenized, and used for HPLC analysis with electrochemical detection. CoQ_9_ was used as an internal standard. Observed CoQ levels were related to the corresponding protein levels of the respective liver homogenate samples (single data not shown). All data (one-sided *t*-test) are means ± SEM of 5 animals in the intervention group (+QH_2_) and control group (−QH_2_), respectively. Protein-related CoQ_10_ levels and CoQ_9_/CoQ_10_ ratio of ubiquinol-10-supplemented and control animals are depicted in Figures 3(a) and 3(b), respectively.

To test putative effects of CoQ_10_ on miRNA 146a regulation in a more physiological manner, liver tissues of QH_2_-supplemented C57BL6/J mice were used. However, to mediate these effects, a tissue-specific accumulation of CoQ_10_ is essential. Therefore, total CoQ_10_ levels were determined in liver homogenate samples of QH_2_-supplemented and control mice. As shown in Figure 3(a), total CoQ_10_ levels increased about 12-fold (*P* = .0193) in liver tissues of QH_2_-supplemented mice when related to control samples. Because CoQ_9_ is the predominant CoQ form in rodents [18], CoQ_9_ was used as an internal standard for HPLC-analysis. Thus, the CoQ_9_ level was not significantly changed between treatment and control group (*P* = .51, data not shown). Accordingly, the CoQ_9_/CoQ_10_ ratio was significantly different between groups, corresponding to a 6.5-fold increase in control tissue samples (*P* = .0019, Figure 3(b)). In general, all HPLC measurements were related to protein levels in the respective tissue homogenate samples. In summary, we could significantly increase the CoQ_10_ concentration in liver tissues of ubiquinol-10-supplemented mice. An effective uptake of exogenously applied CoQ_10_ in liver tissues of rodents has been already described earlier [19].

### 3.4. Reduced Expression Levels of miR-146a in Liver Tissues of Ubiquinol-10-Supplemented Mice

**Figure 4:**
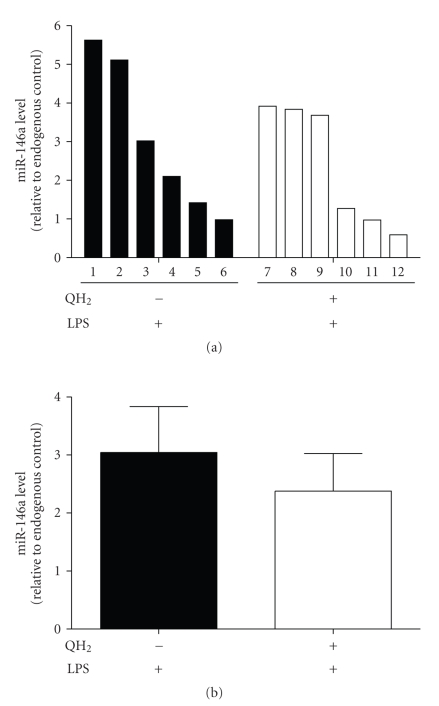
Effect of ubiquinol-10 supplementation on miR-146a expression in liver samples of LPS-treated mice. Mice were fed with ubiquinol-10 (250 mg/kg/d) or a respective control diet (no ubiquinol-10 supplement) for 7 days. Afterwards, a sublethal intraperitoneal injection of LPS (1 mg/kg BW) was administered for further 4 hours Thereafter, liver samples were collected, homogenized, and total RNAs including miRs were isolated as described by the manufacturer's instructions. Subsequently, miRs were converted to cDNA and assayed by the TaqMan-based qRT-PCR. Observed miR-146a expression levels of each animal were related to the corresponding levels of the endogenous control (snoRNA202). Data (two-sided *t*-test) are means ± SEM of 6 animals in the intervention group (+QH_2_/+LPS) and control group (+LPS), respectively. miR-146a expression levels are depicted as single bars of each animal (Figure 4(a)) or as total mean ± SEM of each group (Figure 4(b)).

To test the relevance of a putative ubiquinol-10-dependent downregulation of LPS-inducible miR-146a expression in vivo, we used mice receiving either a diet enriched with ubiquinol-10 (QH_2_, 250 mg/kg/d) or a respective control diet for seven days. This dosage is in accordance with previous CoQ_10_ studies in mice [20–22]. Thereafter, an intraperitoneal injection of lipopolysaccharide (LPS, 1 mg/kg BW) was administered for further 4 hours. In contrast to the control non-LPS-injected mice (no detectable TNF-*α* levels), TNF-*α* levels increased significantly in the serum of LPS-treated animals (612.46 ± SD). Finally, mice were sacrificed and livers were collected for miR isolation and determination of miRNA-146a expression levels. As shown in Figure 4(a), the LPS-induced expression level of miR-146a is generally lower in animals supplemented with ubiquinol-10. In average, ubiquinol-10 reduces the LPS-induced miR-146a expression to 78.12 ± 21.25% (+QH_2_/+LPS) when compared to control animals (−QH_2_/+LPS) (Figure 4(b)). This effect was statistically not significant but was consistent to those obtained in cell culture experiments.

Regulation of miRs by certain compounds or nutrients is of general interest, because this class of noncoding RNAs is involved in central biological processes such as development, inflammation and innate immunity, and signalling networks [1, 23]. So far, only a small number of environmental modulators of miR expression have been identified. Expression levels of several miRs (i.e., miR-15a/b, miR-16, miR-107) are regulated by retinoic acid-induced differentiation in human acute promyelocytic leukemia patients and cell lines [24]. Compounds such as sulphate, phosphate, and amino acids regulate the expression of special miRs in plants [25] and human liver cells [26]. Toll-like receptor (TLR) ligands such as LPS induce the expression of miR-146a significantly in human monocytes/macrophages [*7*].

## 4. Conclusion

Here we identified ubiquinol-10 as a putative modulator of miR-146a expression. Preincubation of THP-1 cells with ubiquinol-10 reduced the LPS-induced expression level of miR-146a. These results are consistent to our in vivo data, where the expression of miR-146a was reduced in liver samples of mice supplemented with ubiquinol-10 before LPS-injection (+QH_2_/+LPS) when compared to control animals (+LPS). Although the observed effects are statistically not significant, we postulated a fine-tuning mechanism of ubiquinol-10 on the inflammatory response via a moderate reduction of miR-146a expression. According to the superior function of miR-146a in the inflammatory response, the observed moderate reduction of its expression by ubiquinol-10 seems to be desirable based on the following mechanisms. First, the LPS induced upregulation of miR-146a in human monocytes/macrophages functions as a negative regulator of the innate immune response because miR-146a targets TRAF6, a regulator protein within the TLR-signalling pathways involved in the formation and accumulation of reactive oxygen species [7]. Second, we have recently shown that ubiquinol-10 lowers the LPS-stimulated release of some proinflammatory cytokines and chemokines relevant in inflammatory processes [27]. The observed effects were comparable to those of the potent and characterized antioxidants N-acetyl-cysteine (NAC) or pyrrolidine-dithiocarbamate (PDTC) [12, 14, 27]. Moreover, mir-146a has been shown to be induced by proinflammatory cytokines such as TNF-*α*, interleukin 1-beta (IL-1*β*), and TLRs [7, 28, 29]. miR-146a was also detected in tissues related to inflammatory diseases including, for example, synovial fibroblasts and rheumatoid synovial tissue [28]. Thus, we conclude that ubiquinol-10 reduces both the secretion of proinflammatory agents and the expression of the anti-inflammatory miR-146a. As a consequence, ubiquinol-10 acts as an anti-inflammatory compound but perpetuates the essential inflammatory response via moderate reduction of miR-146a expression. This dual effect could be due to the radical scavenging activity of ubiquinol-10 since reactive oxygen species are involved in the TLR-signalling pathways.

In conclusion, the consistent in vitro and in vivo data suggest that ubiquinol-10 may fine-tune the inflammatory response via moderate reduction of miR-146a expression.

## Figures and Tables

**Table 1:** Concentration and redox state of CoQ_10_ in the human monocytic cell line THP-1 after incubation with various concentrations of ubiquinol-10 for 24 hours. Data are given as means ± SD of two independent measurements performed in duplicate.

Extracellular		Cellular	
*μ*M ubiquinol-10	pmol CoQ_10_/10^6^ cells	% ubiquinol-10	*μ*M ubiquinol-10^((a)-(b))^
0, medium control	25.62 ± 4.42	74.40 ± 0.14	4.56
0, vehicle control	23.07 ± 0.46	74.70 ± 1.56	4.12
0.1	24.68 ± 2.01	75.25 ± 0.35	4.44
1.0	26.48 ± 3.36	79.35 ± 0.07	5.03
10.0	72.35 ± 1.14	86.10 ± 0.71	14.90
100	627.46 ± 138.08	90.35 ± 1.48	140.35

^(a)^Suggested diameter of monocytes: 20 *μ*m ^(b)^Related to mean values.
